# Inventory of lice of mammals and farmyard chicken in North-eastern Algeria

**DOI:** 10.14202/vetworld.2018.386-396

**Published:** 2018-03-30

**Authors:** Mohamed Nadir Meguini, Souad Righi, Fayçal Zeroual, Khelaf Saidani, Ahmed Benakhla

**Affiliations:** 1 Department of Veterinary Sciences, Chadli Bendjedid University, El Tarf, Algeria; 2 Institute of Veterinary and Agronomic Sciences, Mohamed Cherif Messaadia University, Souk-Ahras, Algeria; 3 Institute of Veterinary Sciences, Saad Dahlab University, Blida, Algeria

**Keywords:** boars, farmyard chickens, lice, North-eastern Algeria, ruminants

## Abstract

**Background and Aim:**

Lice are permanent ectoparasites, extremely specific to their hosts. Their great importance in veterinary medicine remain significant, they can cause their direct pathogenic actions like irritability, dermatitis, anemia, decreased weight gain, and milk production. The purpose of this work was to made the first time an inventory of mammalian lice in North-eastern Algeria.

**Materials and Methods:**

Our survey of lice infestation was conducted on several animal species from five provinces of North-eastern Algeria. A total of 57 cattle, 83 sheep, 77 goats, 111 wild boars, and 63 farmyard chickens were examined. The collection of lice was carried out much more in mammals and chickens during the winter period. Lice were collected either manually or using brushing and kept in flasks containing 70% ethanol. The identification of lice was achieved in the laboratory using a binocular loupe.

**Results:**

Concerning cattle, 63% and 27% of those examined subjects from Souk-Ahras and Guelma study areas, respectively, were carriers of lice. *Damalinia bovis* was the louse most frequently found on cattle in these two regions. Three other species were identified in Souk-Ahras: *Haematopinus eurysternus* (25%), *Linognathus vituli* (10%), and *Solenopotes capillatus* (5%). Regarding sheep, 39% and 24% of examined animals in Souk-Ahras and Guelma, were carrying lice. *Damalinia ovis* was the most frequently encountered lice on sheep in both regions. *Linognathus ovillus* also was identified in Souk-Ahras, representing 0.3% of the collected lice. Concerning goats, 53% and 30% of examined animals in Souk-Ahras and Guelma, were parasitized of lice. Two species of lice were found: *Damalinia caprae* and *Linognathus africanus*. For farmyard chickens, 69% and 100% of the farmyard chicken in Souk-Ahras and Mila were parasitized by lice, respectively. *Menopon gallinae* was the most frequently encountered louse in farmyard chicken in both regions. Eight other species were identified in Mila and four other species only in Souk-Ahras. Finally, 25% and 28% of the wild boars in Annaba and El Tarf were parasitized by lice, respectively. *Haematopinus suis* was the only species found on wild boars in both regions.

**Conclusion:**

These results are to be taken into account for lice control schemes and louse-borne diseases.

## Introduction

Pediculosis, a skin infection of warm-blooded animals, is an important economic problem of many species of livestock and poultry. Lice have been considered as one of the responsible parasites for skin rejection at tanneries due to a skin defect as a result of itching leading to scratching and rubbing due to feeding behavior of lice [1-3].

Five thousand parasitic lice species, allocated to four suborders, have so far been described. The sucking lice, which feed exclusively on blood of eutherian mammals, belong to the suborder Anoplura [4] while the chewing lice, which infest birds and mammals mainly and feed on feathers, dead skin, blood, or secretions, belong to the three remaining suborders: Amblycera, ischnocera, and Rhynchophthirina [5]. Sucking lice (Phthiraptera: Anoplura), permanent and host-specific ectoparasites of eutherian mammals, cause economic losses in livestock through weight loss, hide damage, and mild to severe anemia. Moreover, as vectors, they are capable of transmitting pathogens such as viruses, bacteria, fungi, and protozoa to susceptible hosts [6]. Hornok *et al*. [7], after proving that *Anaplasma* spp. could be transmitted by *Linognathus vituli*, *Linognathus stenopsis*, and *Haematopinus suis*, suggested that louse infestation of domestic animals should deserve more attention, and lice should be counted among the broad range of potential vectors of arthropod-borne pathogens.

In the past, louse used to be controlled through various insecticides [8,9], but recently, new strategies for their control have been developed [8]. For example, lice on cattle have effectively been treated with a variety of insecticide chemistries, including topically applied formamidines, organophosphates, and synthetic pyrethroids, as well as topical or injectable macrocyclic lactones [10].

Nowadays, the availability of louse genome opens new promising perspectives in understanding their biology and their vector competence, which could lead to more efficient and better ways of louse control [11].

So far, apart from chicken louse, no other animal louse infestation has been studied in Algeria. This study is a preliminary investigation on the morphological identification of different lice of mammals and birds in far North-eastern Algeria.

## Materials and Methods

### Ethical approval

This study does not require the approval of the Institute Animal Ethics Committee. However, samples were collected as per standard sample collection procedure without harm to the animals.

### Study area

**Figure-1:**
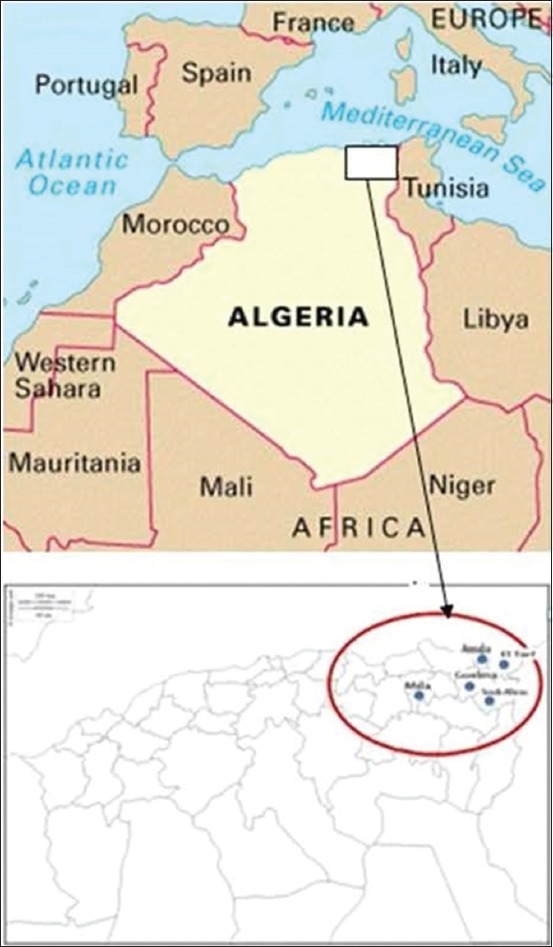
Location of the study areas (Souk-Ahras, Guelma, Mila, El Tarf, and Annaba) on the map of Algeria.

This study was carried out in five provinces from North-eastern Algeria (Souk-Ahras, Guelma, El Tarf, Annaba, and Mila) (Figure-1). The province of Souk-Ahras, consisting of three different zones (the northern zone, median, and south). The northern zone is characterized primarily by mountains, cold, dry climate, and heavy rainfall exceeding 700 mm/year, with extensive breeding. The median zone consists of plains with a subhumid climate and a pluviometry inferior to 700 mm/an, the breeding in this zone is semi-intensive one. The south zone is represented by large areas with a semi-arid climate, hot and dry, and low rainfall levels, i.e., <400 mm/year, characterized by the sheep and goat farming [12].

The province of Guelma is characterized by a diversified relief with important forests. This relief is composed of mountains (37.8%), plains and plateaus (27.2%), and hills and piedmont (26.3%). The territory of the Guelma is characterized by a subhumid climate in the center and the north and semi-arid to the south. This climate is mild and wet in winter and hot in summer. The temperatures vary from 4°C in winter to more than 35°C in summer, with an average of 17.3°C. The pluviometry ranges from 400 to 500 mm/year in the south to nearly 1000 mm/year in the North.

El Tarf province has a Mediterranean climate, characterized by a great pluviometry, a hot summer, and a mild winter temperature. It is one of the most watering areas in the North of Africa. This province is so close to the sea; this one plays the role of the condenser of tropical air masses and humid areas (Tonga Lake, Oubeira Lake, and Mallah Lake) undergo evaporation, which is sometimes intense because of getting sunshine; they are the origin of high atmospheric humidity [13].

Annaba province is a coastal region enjoys a Mediterranean climate. It is known for its long hot, humid summers. The winters are mild and humid, and rains are abundant. The relief of this province consists of mountains (52.16%), hills and foothills (25.82%), and plains (18.08%) [14].

Mila province is characterized by a diversity of landscapes including mountain massifs in the north part of the province with hills and Piedmont and in the central part of the province high plains. The climate is characterized by hot and dry summers, cold and wet winters, with precipitation varying from north to south from 350 to 700 mm/year, according to zones. The province shelters the biggest dam of water at the national level, namely, the Beni Haroun dam, which provides most drinking water to the major part of Coastal Algeria as well but also water of irrigation [15].

### Animals

The study has involved several animal species including cattle, goats, sheep (provinces of Guelma and Souk Ahras), wild boar (provinces of Annaba and El Tarf), and farmyard chickens (provinces of Mila and Souk Ahras). For cattle, sheep, and goats, 4 and 12 farms were visited in Guelma and Souk Ahras provinces, respectively. These farms were selected randomly with a number of animals examined totaling 11 cattle, 21 sheep, and 30 goats in Guelma province and 46 cattle, 62 sheep, and 47 goats in Souk Ahras province. For free-range chickens, the study was conducted in 4 and 5 farms from Mila and Souk Ahras provinces, respectively. A total of 63 checked were examined (31 and 32 from Mila and Souk Ahras provinces, respectively). Finally, a total of 111 wild boars were examined (72 and 39 from Annaba and El Tarf, respectively).

### Sample collection

The collection of lice was carried out much more in mammals and chickens during the winter period, which is the period of activity of these insects [16].

For ruminants, the collection of lice in the Souk-Ahras region was carried out during October 2014 to April 2015 (autumn, winter, and spring). In the Guelma region, the collection was conducted only during October-November 2014. Cattle, sheep, and goats were carefully checked (paying particular attention to examine the different parts of the body surface) to look for the presence of lice. Once spotted, brushing with a comb was carried out for a period varying between 10 and 30 min depending on the intensity of infestation. The collected lice were stored in numbered tubes containing 70% ethanol.

For chickens, lice were collected randomly, at a frequency of 4 chickens per month between September 2015 and April 2016 in Souk-Ahras province, and per month between December 2015 and March 2016 in Mila province. For each chicken, the following manipulations were performed.

To determine the sites of different species of lice, different parts of the body were examined with meticulous care: The head and neck, the feet, the wing feathers, the belly feathers, the rump, and tail feathers.

For lice hard to be captured, the chicken was sprinkled with an insecticide then put in a small place on a sampling surface during 60 min. During this period, most parasites die and fall. Then, feathers were ruffled while the chicken was kept over the sampling surface to salvage the remaining parasites [17]. Parasites were collected using a slim line-rimmed pair of pliers and kept in flasks containing 70% ethanol.

Lice of wild boars were collected from April 2011 to March 2013. The presence of these hematophagous ectoparasites was noticed in this study over a period spreading out from November to March.

Their collection was in most cases, as difficult as that of ticks, because of their small-size (≤5 mm), in addition these insects hide easily between the hairs, as well as they have often a confused color with those, specially since the fur of captured wild boars are most often soiled with mud. Once they are discovered, the lice move to the base of the bristles that are thick and often dark black at the base otherwise they are moving to another location adjacent more adequate and more protective against threats such as the parties containing folds of skin. Once identified, lice were harvested with gloved hands or using tweezers, to be retained in tubes filled with 70% ethanol with identifications particulars (nature of the levy, animal, sex, age, code, date of levy, body region, and capture region) [18].

### Louse identification

The identification of lice was achieved in the laboratory of parasitology of the agro-veterinary Institute (University of Souk-Ahras) as well as in the Laboratory of Parasitology of the Department of Veterinary Sciences (University of El Tarf) using a binocular loupe. The identification of the lice (genus and species) of mammals has been based on the observation of morphological characters established by Wall and Shearer [9] and Pajot [19]. By contrast, for the identification of free-range chicken lice, this last is based on the use of the keys to Emerson [20], Tuff [21], and Furman and Catts [22].

### Statistical analysis

Data normality of the dependent variable (the parasite burden or number of lice parasitizing each animal) was performed using Shapiro–Wilk normality test. χ^2^ test (Chi-square test or Pearson’s test) was applied to compare the percentages or prevalence of lice infestation. Analysis of variance was implemented to compare levels of distinct factors (categories of age, provinces, lice genus, lice species, and month of examination). Before employing any nonparametric test, the data related to the intensities of infestation were transformed by either the logarithmic or the square root function to meet the normality and homogeneity of variances conditions. The Kruskal–Wallis test was used for non-normal data for comparing the parasite loads of lice collected on ruminants, free range chickens as well as the wild boar in the regions of Souk-Ahras, Guelma, Mila, Annaba, and El Tarf.

All the analyses were achieved by Statistica 10, SPSS (Statistical Package for the Social Sciences) version 22, and the open source Software R (R core team 2015) [23]. Whatever the test used, a difference is declared as significant if p≤0.05.

## Results

### Relative abundance and parasitic burden on ruminants (in both Souk-Ahras and Guelma study areas)

29 out of 46 and 3 out of 11 examined cattle were found to be infested with lice in Souk-Ahras and Guelma, respectively. In total, 1901 adult parasites, whose 1590 (84%) in Souk-Ahras and 311 (16%) in Guelma, were collected (Table-1). The lice collected in Guelma belonged entirely to the *Damalinia bovis* (Ischnocera) species, whereas in Souk-Ahras, the latter accounted for 58%, and where three other species (all Anoplura) also were found: *Haematopinus eurysternus* for 25%, *L. vituli* for 10%, and *Solenopotes capillatus* for 5% of all the lice collected. The parasitic load of *D. bovis* was 103.6 in Guelma and 66.5 in Souk-Ahras.

**Table-1:** Distribution of lice of cattle in the two areas of study (Northeastern of Algeria).

Lice species	Souk - Ahras				Guelma			
	
	Number of infested cattle and rate infestation (%)	Lice number	Relative abundance (%)	Parasitic burden	Number of infested cattle and rate infestation (%)	Lice number	Relative abundance (%)	Parasitic burden
*D. bovis*	14 (30.4)	931	58.5	66.5	3 (27.3)	311	100	103.6
*H. eurysternus*	6 (13)	402	25.3	67				
*L. vituli*	5 (10.8)	172	10.8	34.4				
*S. capillatus*	4 (8.7)	85	5.3	21.2				
Total	29	1590	83.6	54.8	3	311	16/4	103.6

D. bovis=Damalinia bovis, H. eurysternus=Haematopinus eurysternus, L. vituli=Linognathus vituli, S. capillatus=Solenopotes capillatus

24 out of 62 in Souk-Ahras and 5 out 21 in Guelma examined sheep were infested with lice. In total, 735 adult parasites, whose 689 Souk-Ahras (94%) and 46 in Guelma (6%) were collected (Table-2). In Guelma, the lice collected were all of the ischnocera *Damalinia ovis* species but in Souk-Ahras, the latter represented around 99.7% and other species of sucking lice (Anoplura) *Linognathus ovillus* represented the rest, i.e., 0.3%. The parasitic load by *D. ovis* was 9.2 in Guelma and 29.9 in Souk-Ahras.

**Table-2:** Distribution of lice of sheep in the two areas of study (North-eastern of Algeria).

Lice species	Souk - Ahras				Guelma			
	
	Number of infested sheep and rate infestation (%)	Lice number	Relative abundance (%)	Parasitic burden	Number of infested sheep and rate infestation (%)	Lice number	Relative abundance (%)	Parasitic burden
*D. ovis*	23 (37.1)	687	99.7	29.9	5 (23.8)	46	100	9.2
*L. ovillus*	1 (1.6)	2	0.3	2				
Total	24	689	93.7	28.7	5	46	6.3	9.2

D. bovis=Damalinia bovis, L. ovillus=Linognathus ovillus

Data of Table-3 show that 25 out of 47 in Souk-Ahras and 9 out 30 in Guelma examined goats were found to be infested with lice. In total, 356 adult parasite, whose 251 in Souk-Ahras (70.5%) and 105 (29.5%) in Guelma (Table-3) were collected. Among the lice collected in Guelma, the chewing louse *Damalinia caprae* represented 71% and the sucking louse *Linognathus africanus* represented 28%. In Souk-Ahras, however, *L. africanus* predominated (62%) and *D. caprae*, accounted for 37% of the collected lice. The parasitic load of *L. africanus* was 10.5 in Souk-Ahras and 5 in Guelma, and the parasitic load of *D. caprae* was almost similar in both areas studied.

**Table-3:** Distribution of lice of goats in the two study areas (North-eastern of Algeria).

Lice species	Souk-Ahras				Guelma			
	
	Number of infested goats and rate infestation (%)	Lice number	Relative abundance (%)	Parasitic burden	Number of infested goats and rate infestation (%)	Lice number	Relative abundance (%)	Parasitic burden
*D. caprae*	10 (21.3)	94	37.5	9.4	7 (23.3)	75	71.4	10.7
*L. africanus*	15 (31.9)	157	62.5	10.5	6 (20)	30	28.6	5
Total	25	251	70.5	10	9	105	29.5	11.6

D. caprae=Damalinia caprae, L. africanus=Linognathus africanus

### Relative abundance and parasitic burden in farmyard chickens (in both Souk-Ahras and Mila study areas)

Data of Table-4 show that 22 out of 32 in Souk-Ahras and 31 over 31 in Mila of the farmyard chickens examined were infested with lice. In total, 5212 adult parasites, whose 2001 in Souk-Ahras (38%) and 3211 in Mila (62%) (Table-4) were collected.

**Table-4:** Distribution of lice of chickens farmers in the two study regions (North-eastern of Algeria).

Lice species	Mila				Souk-Ahras			
	
	Number of infested chickens and rate infestation (%)	Lice number	Relative abundance (%)	Parasitic burden	Number of infested chickens and rate infestation (%)	Lice number	Relative abundance (%)	Parasitic burden
*M. gallinae*	31 (100)	1875	58.4	60.5	22 (68.7)	1583	79.1	71.9
*M. stramineus*	24 (77.4)	1128	35.1	47	2 (6.2)	16	0.8	8
*L. caponis*	3 (9.7)	3	0.09	1	7 (21.8)	78	3.8	11.1
*C. meleagridis*					6 (18.7)	317	15.8	52.8
*G. gigas*	1 (3.2)	1	0.03	1	3 (9.3)	7	0.35	2.3
*G. gallinae*	20 (64.5)	186	5.8	9.3				
*M. pallidulus*	4 (12.9)	8	0.25	2				
*G. dissimilis*	3 (9.7)	3	0.09	1				
*C. heterographus*	1 (3.2)	1	0.03	1				
*M. cornutus*	4 (12.9)	6	0.19	1.5				
Total	31	3211	61.6	100	22	2001	38.4	90.9

M. gallinae=Menopon gallinae, M. stramineus=Menacanthus stramineus, L. caponis=Lipeurus caponis, C. meleagridis=Chelopistes meleagridis, G. gigas=Goniodes gigas, G. gallinae=Goniocotes gallinae, M. pallidulus=Menacanthus pallidulus, G. dissimilis=Goniodes dissimilis, C. heterographus=Cuclotogaster heterographus

In Mila, the amblycera *Menopon gallinae* accounted for 58.4% of the lice collected. The rest comprised eight other species: *Menacanthus stramineus* (35%), *Menacanthus pallidulus* (0.25%), *Menacanthus cornutus* (0.19%), *Goniocotes gallinae* (5.8%), *Goniodes gigas* (0.03%), *Goniodes dissimilis* (0.09%), *Cuclotogaster heterographus (*0.03%), and *Lipeurus caponis* (0.09%).

Among the five lice species identified in Souk-Ahras, the amblycera *M. gallinae* was the most predominant (79%). The four other lice species collected: *Chelopistes meleagridis*, L. *caponis*, *M. stramineus*, and *G. gigas* accounted for 15.8%, 3.8%, 0.8%, and 0.35%, respectively.

The parasitic load of *M. gallinae* and that of *L. caponis* were higher in Souk-Ahras than in Mila: 72 versus 60.5 and 11 versus 1, respectively.

On the contrary, the parasite load of *M. stramineus* was higher (47) in Mila than in Souk-Ahras (8).

### Relative abundance and parasitic load of the wild boar (in both Annaba and El Tarf study areas)

Eighteen out of 72 in Annaba and 11 out of 39 in El Tarf of wild boars examined were lice infested. In total, 434 adult parasites, whose 272 in Annaba (63%) and 162 in El Tarf (37%) (Table-5) were collected. *H. suis* was the sole lice species collected in both areas with a similar parasitic load (15).

**Table-5:** Distribution of lice of wild boar in the two study regions (North-eastern of Algeria).

Lice species	Annaba				El Tarf			
	
	Number of infested wild boar and rate infestation (%)	Lice number	Relative abundance (%)	Parasitic burden	Number of infested wild boar and rate infestation (%)	Lice number	Relative abundance (%)	Parasitic burden
*H. suis*	18 (16.2)	272	100	15.1	11 (10)	162	100	14.7
Total	18	272	62.7	15.1	11	162	37.3	14.7

H. suis=Haematopinus suis

### Monthly variations of ruminants infestation

**Figure-2:**
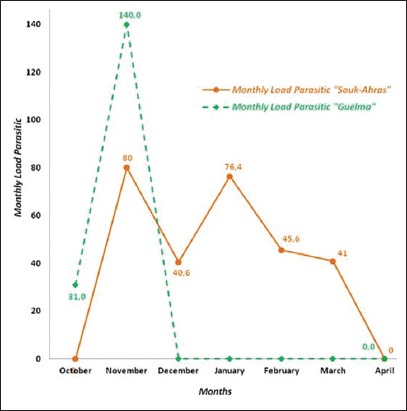
Monthly load parasitic of lice of cattle in the two study regions.

Lice infestation in cattle varied intensively during 5 months in Souk-Ahras and only during 2 months in Guelma (Figure-2). In Souk-Ahras, cattle were more infested between November and March, loading up to 80 lice per animal in November. In Guelma, the heaviest infestation load (140) was recorded in November. The monthly loads of parasitic lice were not significantly different between the two distinct study areas (p>0.05).

**Figure-3:**
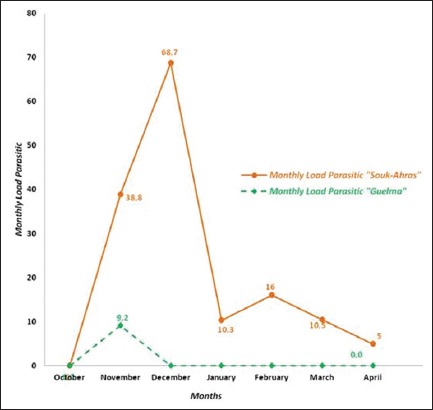
Monthly lice infestation burden in sheep in the two studied regions.

The parasitic lice load variation in sheep was studied during 6 months in Souk-Ahras and only during 1 month in Guelma (Figure-3). In Souk-Ahras, the sheep were heavily infested between November and April with a maximum load of 68.7 lice per sheep recorded in December. In Guelma, the heaviest lice infestation was recorded in November with a load of 9.2 lice per animal. The monthly parasitic burdens were not significantly different between the two areas of study (p>0.05).

**Figure-4:**
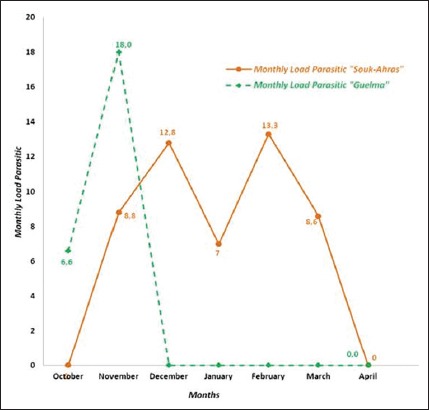
Monthly parasitic loads in goats in the two study regions.

The lice infestation load in goats lasted 5 months in Souk-Ahras and only 2 months in Guelma (Figure-4). In Souk-Ahras, the heaviest infestation was recorded between November and March, with a maximum load of 13.3 lice per goat in February. In Guelma, the heaviest infestation was seen (18) in November.

The monthly parasitic burden was significantly higher in Souk-Ahras than in Guelma (p<0.05).

### Monthly variations of farmyard chickens infestation

**Figure-5:**
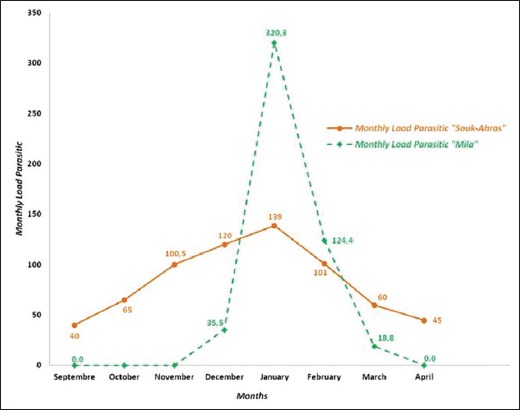
Monthly lice infestation loads in farmyard chickens in the region of Souk-Ahras and Mila.

The lice infestation burden variation in free-range chickens was observed during 8 months in Souk-Ahras and 4 months in Mila (Figure-5). In Souk-Ahras, the chickens were more intensively infested between September and April, with a maximum load of 139 lice per farmyard chicken recorded in January. In Mila, the most intensive infestation was seen during January (320.3). The monthly parasitic loads were not significantly different between the two areas of study (p>0.05).

### Monthly variations of the wild boar infestation

**Figure-6:**
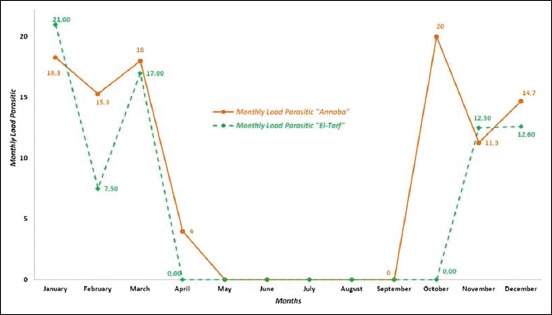
Monthly parasitic burden in wild boar in the region of Annaba and El Tarf.

The parasitic load variation of lice in wild boars was observed during 7 months in Annaba and only 5 months in El Tarf (Figure-6). In Annaba, the infestation was more intensive between January and December, with a maximum load of 20 lice per wild boar recorded in October. In El Tarf, the most intensive load (21 per animal) was seen in January. The monthly parasitic loads were not significantly different between the two areas of study (p>0.05).

## Discussion

### Balance sheet of lice collection

Nearly 63% and 27% of cattle examined in Souk-Ahras and Guelma, respectively, were lice infested. A prevalence of 4.7% and 38.3% was reported in Tunisia [16] and Pakistan [24], respectively. Christensson *et al*. [25] noted that in Europe 29% of the studied animals were lice carriers and similar results were also reported in Scotland and the Netherlands [26,27].

The results reported in the present study may be due to a lack of awareness from the farmers regarding the infestation economic impact that is difficult to estimate.

Nafstad and Gronstol [28] mentioned that lice are a significant problem which remains under-estimated among non-treated herds.

The parasitic average load recorded, as high as 79 lice per animal, is a significant number that may demonstrate the little interest the farmers have as to the use of insecticides to treat these parasites.

*D. bovis*, the most frequently encountered louse in cattle in the two areas studied (chewing lice species or Mallophaga), is a species which remains far more abundant and the more clinically important that can multiply by parthenogenesis, which leads to the very rapid increase in its number.

This species was reported to be predominant in New York [29], Ethiopia [30], Tunisia [16], and Pakistan [24] at a relative abundance of 90%, 23.7%, 4%, and 100%, respectively.

In addition to *D. bovis*, three other species (*H. eurysternus*, *L. vituli*, and *S. capillatus*) were identified in Souk-Ahras. These latter are also among the species of lice encountered in Hungary [7], Tunisia [16], Canada [31], and Bangladesh [32].

*D. bovis* was observed in a high proportion in Souk-Ahras (75% of the lice collected). Climates characterized by dry winters are suitable for the development of this chewing louse, but tropical ones are not [33]. Moreover, in Ethiopia [34] and Pakistan [35], *D. bovis* was not reported. Souk-Ahras seems to be a favorable medium for its development due to its dry winter.

The presence of three other sucking lice species in Souk-Ahras, could be inherent to the ranchers lack of ectoparasites control, and the short period spent collecting lice (2 months) in Guelma, not sufficient enough to cover a great part of the area studied may explain the low prevalence of *D. bovis* as well as the absence of other sucking lice species.

Nearly, 39% and 24% of sheep examined in Souk-Ahras and Guelma, respectively, were lice infested. The parasitic average load was 19 lice/animal.

This infestation could be related to the farmers under-estimation of these ectoparasites, poor husbandry practices and subsequently no insecticides treatment.

*D. ovis* (sheep biting louse) was the most frequently encountered louse species among sheep in the two areas investigated. This species was reported to be predominant in Oromia (Ethiopia) [36] at a relative abundance of 100% and also by Eticha *et al*. [37].

Fekadu *et al*. [38] and Eshetu *et al*. [39] noted, respectively, a relative abundance of 36.1% and 12.07% of this species in three Agro-Ecological districts of southern (Ethiopia) and Wogera district, north Gondar Zone (Ethiopia).

Zangana *et al*. [40] also reported a high relative abundance of this louse in Iraq (75%) and Sao Paulo (Brazil). Madeira *et al*. [41] have reported a relative abundance of 13.8%.

Recall that *D. ovis* requires low temperatures and survive only in small numbers in the summer. *D. ovis* was observed in a higher proportion in Souk-Ahras (94% of the lice collected); its parasitic load was also more important. According to Price and Graham [33], this species remains worldwide distributed.

In addition to *D. ovis*, *L. ovillus* (face lice) has also been found in Souk-Ahras. This species is among the species of lice surveyed in Ethiopia [42-44] and in India [45].

Fifty-three in Souk-Ahras and 30% in Guelma, of examined goats were, respectively, infested by lice. The parasitic load average was 10.8 lice/animal. This infestation is also linked to the under-estimation of these external parasites by farmers and subsequently the non-use of insecticides treatment.

In goats, two species of lice were identified during the present study: *D. caprae* and *L. africanus* (African blue louse). The latter was the most frequently observed on goats in Souk-Ahras. The predominance of this species has been reported in eastern part of Ethiopia [46], at a relative abundance of 7.8% and also at a relative abundance of 60% in Italy [47]. It is to be noted that this species had not been reported for a while in several countries including Spain [48], Hawaii, the Philippines, and India [49], Libya [50], and Australia [51].

In Guelma, *D. caprae* (goat biting louse) was the most frequent louse species encountered. The predominance of this species has been reported in the region of Dehradun (India) [52], at a relative abundance of 79%, in the north of Sinaî (Egypt) [53] at a relative abundance of 31%, and in Gondar Town (Ethiopia) [54], at a relative abundance of 26.12%. This species has a cosmopolitan distribution since it has been reported in many other parts of the world: In the United States, Argentina, Colombia, Brazil, and Cuba [55], in Chile and France [56], Uganda, and South Africa, and in India [57].

Dos Santos *et al*. [58] suggested that adaptation to climate change could be different in the two species of goat lice; this may explain the predominance of *L. africanus* in Souk-Ahras and *D. caprae* in Guelma.

### In farmyard chickens

Nearly 69% and 100% out of the free-range chickens examined in Souk-Ahras and Mila were parasitized by lice, respectively. The parasitic load average was 95 lice/farmyard chicken. This infestation encountered in the traditional farms is linked to the lack of hygiene as well as to that of interest in lice.

*M. gallinae* was the most common louse found on farmyard chickens in both areas investigated. This predominance has also been reported in several other parts of the world: In Penang (Malaysia) [59] with the highest mean abundance (76.7%), El Tarf (Algeria) [13] at a relative abundance of 48%, Himachal Pradesh (India) [60] at a relative abundance of 51%, and in Oaxaca (Mexico) [61] at a relative abundance of 86%.

In addition to *M. gallinae*, eight other species were identified in Mila, namely: *M. stramineus*, *G. gallinae*, *M. pallidulus*, *M. cornutus*, *G. gigas*, *C. heterographus, L. caponis*, and *G. dissimilis*. These latter are among the nine species of lice found in El Tarf [13]. In Souk-Ahras, however, only five louse species were identified: *M. gallinae*, *L. caponis*, *M. stramineus*, *G. gigas, and C. meleagridis*. The latter was not found in Mila. This species has also been reported by Maturano and Daemon [62].

The diversity of louse species in Mila as compared to that of Souk-Ahras can potentially be due to the high humidity which characterizes Mila. Tchedre [63] reported that environmental conditions relating to the poultries in the traditional environment are favorable to the survival and the multiplication of ectoparasites and especially lice.

Murillo and Mullens [64] noted that most of these louse species are probably not of serious economic importance, with the exception of those that feed on blood.

### In wild boar

Nearly 25% and 28% out of the wild boars examined in Annaba and El Tarf were parasitized by lice, respectively. The parasitic load average was 15 lice/animal. *H. suis* (Hog louse) was the only species of louse encountered on the wild boar in the two areas investigated. Hornok *et al*. [7] have also revealed a high relative abundance (100%) in the Northeast of Hungary. Previous studies have reported the existence of *H. suis* in other countries including Turkey [65], Germany [66], Nigeria [67,68], and Kenya [69]. According to Price and Graham [33], *H. suis* has a cosmopolitan distribution and is usually more widespread in temperate climates, recalling, however, that *H. suis* is the only species of lice affecting the pigs and wild boar [70].

### Monthly variations of the infestation

In cattle, lice were present in Souk-Ahras from November to March with a peak in November, and from October to November in Guelma. Gharbi *et al*. [16], during the fall and winter seasons in Nabeul (Tunisia), revealed the presence of lice from September to February. Colwell *et al*. [31] reported a wintry activity of cattle lice in the south of Alberta (USA). According to Franc [71], lice are more abundant in winter among the population of cattle of temperate countries when they are indoors; they decrease in spring and almost disappear in summer. During the warm season, a few individuals survive in protected body parts which sustain the infestation.

In sheep, lice were present in Souk-Ahras from November to April with a peak in December whereas in Guelma they were present only during November. Elsaid *et al*. [72] noted that the amount of fleece and shearing were powerful regulating influences, which can remove most of the population. It also exposes the remaining lice to environmental influences (high skin temperature and solar radiation).

In goats, lice were present in Souk-Ahras from November to March with a peak in February and in Guelma from October to November. YDuring 1-year study in Iran on sheep and goats found that the degree of infestation was the highest in fall and winter and lowest during spring and summer [73].

Regarding the farmyard chickens infestation, lice were present from September to April in Souk-Ahras and from December to March in Mila with a peak in January in both regions. Medjouel *et al*. [13], in El Tarf (Far East of Algeria) over a year study period, found a very important lice load from December to February with a peak in January, and a low load from March to November.

Regarding wild boars infestation, lice were present from October to April in Annaba and from November to March in El Tarf. Hornok *et al*. [7], in the central and the North-eastern Hungary, reported the presence of lice in March and Gipson *et al*. [74], in Kansas (USA), reported their presence from November to February. By contrast, Braae *et al*. [75] noted the presence of lice in the dry season from May to August, in their study in the Mbeya Region, Tanzania.

Franc [71] explained that the fall and winter activity of lice is due to their negative phototaxis since they need a very little heat and that direct light and solar heat are harmful. It is well known that the increase in the temperature of the skin surface of the animals can lead to the death of these parasites.

## Conclusion

The high frequency of lice in ruminants both in Souk-Ahras and Guelma leaves a fear of endemic presence of certain diseases transmitted to these animals by lice. A study not yet published, focusing on the detection by PCR of pathogens vectored by lice in Souk-Ahras and Guelma, was able to detect *Borrelia* spp., *Anaplasma* spp., and *Bartonella* spp.

It would, therefore, be necessary to apply insecticide treatments to ruminants during the period of infestation to prevent these louse-borne diseases. In free range chickens, the association insecticide treatments to the hygiene are crucial to minimize damages and preserve the traditional poultry.

## Authors’ Contributions

MNM and SR have conceived and designed the study (ruminants and farmyard chickens infestation), FZ conceived and designed the study (wild boar infestation), KS has performed statistical analysis, AB performed the study and had specially conceived the farmyard chickens study in Mila. All authors read and approved the final manuscript.
